# Thyroid volume, goiter prevalence, and selenium levels in an iodine-sufficient area: a cross-sectional study

**DOI:** 10.1186/1471-2458-13-1153

**Published:** 2013-12-10

**Authors:** Yang Liu, Hui Huang, Jing Zeng, Chengjun Sun

**Affiliations:** 1 Department of Endocrinology, West China Hospital, Sichuan University, No.37 Guoxue Lane, Chengdu, Sichuan Province 610041, China; 2 Department of Physics and Chemistry, School of Public Health, Sichuan University, Chengdu 610041, China

**Keywords:** Iodine, Selenium, Thyroid hormones, Thyroid volume, Thyroid goiter, Thyroid disease, Cross-sectional study

## Abstract

**Background:**

Selenium (Se) is a necessary element for the biosynthesis of thyroid hormones. We investigated the relationship between selenium status, thyroid volume, and goiter in a cross-sectional study in an iodine-sufficient area.

**Methods:**

We selected residents of Chengdu (over 18 years old and living in the city for more than 5 years) using a stratified cluster sampling technique. Fifteen hundred subjects were selected for the study, which involved a questionnaire survey, physical examination, thyroid ultrasound, serum thyroid function test, and determination of serum selenium level. Thyroid volume was calculated from the thickness, width, length, and a corrective factor for each lobe. Ultimately, 1,205 subjects completed the investigation and were included in our study. Additionally, 80 school-age children were selected to provide urine samples for urinary iodine analysis. We analyzed the data using appropriate nonparametric and parametric statistical tests.

**Results:**

The median urinary iodine value was 184 μg/L in school-age children, indicating iodine sufficiency. The median serum selenium level of the 1,205 subjects was 52.63 (interquartile range [IQR] : 40.40-67.00) μg/L. The median thyroid volume was 9.93 (IQR: 7.71-12.57) mL; both log-transformed serum selenium and log-transformed thyroid volume were Gaussian distributions (*P* = .638 and *P* = .046, respectively). The prevalences of goiter and thyroid nodules were 8.8% and 18.6%. The prevalences of positive thyroid autoantibodies, thyroperoxidase autoantibodies and thyroglobulin autoantibodies were 16.7%, 12.0%, and 11.1%, respectively. In the general linear regression model, there were positive associations between serum selenium and age, and body mass index. We found no association between serum selenium and thyroid-stimulating hormone. In simple linear regression analyses, we found no association between thyroid volume and serum selenium. There were no significant differences in serum selenium between persons with or without goiter. Serum selenium was not a risk factor for goiter.

**Conclusion:**

In our study population, serum selenium was neither associated with thyroid volume nor with goiter in an iodine-sufficient area. More studies should be conducted by following non-goitrous persons over time and monitoring their selenium status.

## Background

Both iodine and selenium are essential micronutrients for proper thyroid function. Iodine is a main component of the thyroid hormones thyroxine (T4) and triiodothyronine (T3). The trace element selenium is a component of selenocysteine and has an important role in normal thyroid hormone metabolism; it is involved in the catalysis of all known selenoenzymes (e.g., iodothyronine deiodinases and glutathione peroxidase). Compared with other organs, the thyroid has the highest selenium concentration [1]. Thyroxine is produced exclusively in the thyroid gland, and it may be converted to biologically active triiodothyronine by the iodothyronine 5′-deiodinases in the peripheral tissue. Hypothyroidism or hyperthyroidism will occur when the thyroid gland cannot provide appropriate thyroid hormones. On the other hand, the antioxidant selenium protein glutathione peroxidase can protect the thyrocytes from any excess hydrogen peroxide, which is produced during thyroid hormone biosynthesis [2].

Different populations vary considerably in serum selenium concentrations. Selenium levels can be influenced by a number of factors such as ethnicity, dietary habits and individual bioavailability of selenium [3]. For clinical researchers, it is difficult to compare studies on serum selenium and thyroid goiter. Researchers have investigated the relationship between thyroid volume, goiter, and serum selenium, but some have not taken into account the influence of iodine nutrition status on thyroid volume. Some trials have investigated populations with concurrent iodine deficiency [4-6], while others have failed to describe the iodine status of a population [7]. However, iodine nutritional status in a population seems to be a key factor in maintaining normal thyroid volume and function [8,9]. Therefore, selenium’s effect on goiter in iodine-deficient areas is not yet clear. Until now, there were also few data on the influence of selenium on thyroid volume in iodine-sufficient areas. We investigated the relationship between thyroid volume, goiter, and serum selenium**.**

## Methods

All subjects in this study came from a community in Chengdu (1 of 10 cities selected for epidemiological investigations of thyroid disease in China). Fifteen hundred residents who had lived in Chengdu for at least 5 years and were more than 18 years old were selected using a stratified cluster sampling technique. The response rate was 80.3%. The 1,205 subjects that completed the investigation were included in this analysis. The exclusion criteria for the study were as follows: (1) Women who were pregnant or less than one year postpartum; (2) subjects taking glucocorticoids, dopamine, or dobutamine; (3) subjects taking antiepileptic drugs (phenytoin, carbamazepine, or others); (4) subjects suffering from adrenocortical insufficiency, renal insufficiency, or other serious systemic disease or chronic wasting disease; or (5) a person receiving amiodarone or an iodine-containing contrast agent within the past 6 months.

All the participants were asked to complete a self-assessment questionnaire that included demographic data, reproductive history, history of smoking and previous thyroid disease, and family history of thyroid disease. Height, weight, and blood pressure were measured in all participants. Fasting blood samples were collected and centrifuged at 3,000 revolutions per minute for 5 minutes. Sera were decanted for storage at −20°C until assayed. Fasting urine specimens from 80 school-age children from a school in the community were collected and frozen (−20°C).

A thyroid ultrasonography was performed with a 7.5 MHz (LOGIQ 500, GE Healthcare, China). Thyroid volume was calculated by multiplying the thickness, width, length, by a corrective factor (0.479) for each lobe [10]. The diameter of thyroid nodules was measured and recorded. The thyroid echo pattern was classified as isoecho, hyperecho, hypoecho, and heterogeneous echo.

The study was approved by the ethics committee of West China Hospital, Sichuan University. Participants signed a consent form after being informed verbally about the study.

### Laboratory methods

Thyroid function and thyroid autoantibodies were measured using chemiluminescence immunoassay kits (Roche Kit, Cobas-e601 analyzer). Intraassay and interassay coefficient of variation were all less than 5%. Thyroperoxidase autoantibody (TPOAb), thyroglobulin autoantibody (TgAb), and thyroid stimulating hormone (TSH) were measured in all participants. If TSH was <0.71 mU/L, the free thyroxine (FT4) and free triiodothyronine (FT3) were also measured in the same sample. If TSH was >6.25 m U/L, then only FT4 was measured. Serum selenium was determined by hydride generation atomic fluorescence spectrometry (HG-AFS) using a method described elsewhere [11]. Serum was digested in a mixture of concentrated HNO3 and HC1O_4_ (V: V = 4:1) first and then determined by HG-AFS. During the process of digestion, the temperature was kept below 180°C to prevent selenium from volatilizing. Digestion was continued at 180°C until the solution became clear. The solutions of HCL (V:V = 1:20) and KBH4 (M:V = 1.5:100) diluted by deionized water were used as the carrier liquid and reducing agent, respectively. The median urinary iodine was measured by As-Ce catalytic chromatography. Laboratory reference values were 12 to 22 pmol/L for FT4, 3.1 to 6.8 pmol/L for FT3, 0.71 to 6.25 mU/L for TSH, <34 IU/mL for TPOAb, and <115 IU/mL for TgAb.

### Diagnostic criteria for thyroid disease

Clinical hyperthyroidism (overt thyrotoxicosis) was defined as a TSH < 0.71 mU/L and an FT4 >22 pmol/L, and/or a TSH <0.71 mU/L and an FT3 > 6.8 pmol/L. Subclinical hyperthyroidism (subclinical thyrotoxicosis) was based on a normal FT4 and FT3, and a TSH <0.71 mU/L. Clinical hypothyroidism (overt hypothyroidism) was defined as a TSH >6.25 mU/L and FT4 <12 pmol/L. Subclinical hypothyroidism was based on a normal FT4 and TSH >6.25 mU/L.

Goiter was defined as a thyroid volume exceeding 18.8 mL for males and 14.4 mL for females. The cut-off levels were derived from the mean (+2 SD) thyroid volume in 597 subjects (315 males and 282 females) without thyroid dysfunction, without a previous thyroid disease, without a family history of thyroid disease, without positive thyroid autoantibodies, and without goiter or nodules on ultrasonography [12].

### Statistical methods

Data processing was done with the SPSS version 16.0 (Chicago, SPSS Inc.). Laboratory values were reported as mean ± SD, percentage, median, and interquartile range, where approximately. Normality of the data distribution was assessed with the Kolmogorov-Smirnov test. For continuous variables, nonparametric statistics (Mann–Whitney or Kruskal-Wallis), and parametric statistics (*t* test) were all used, as appropriate. Group differences in the numbers of subjects were analyzed using the chi-square test. The relationship between serum selenium concentration and thyroid volume was investigated by simple and multivariate linear regression, with log-transformed thyroid volume as the dependent variable. The relationship between selenium status and thyroid goiter was examined by univariate and multivariate logistical regression. Apart from serum selenium levels, the following variables were included in the models: age, gender, smoking, body mass index (BMI), TSH concentrations, thyroid nodules, and thyroid echogenicity. The level of significance was set at 5%.

**Figure 1:**
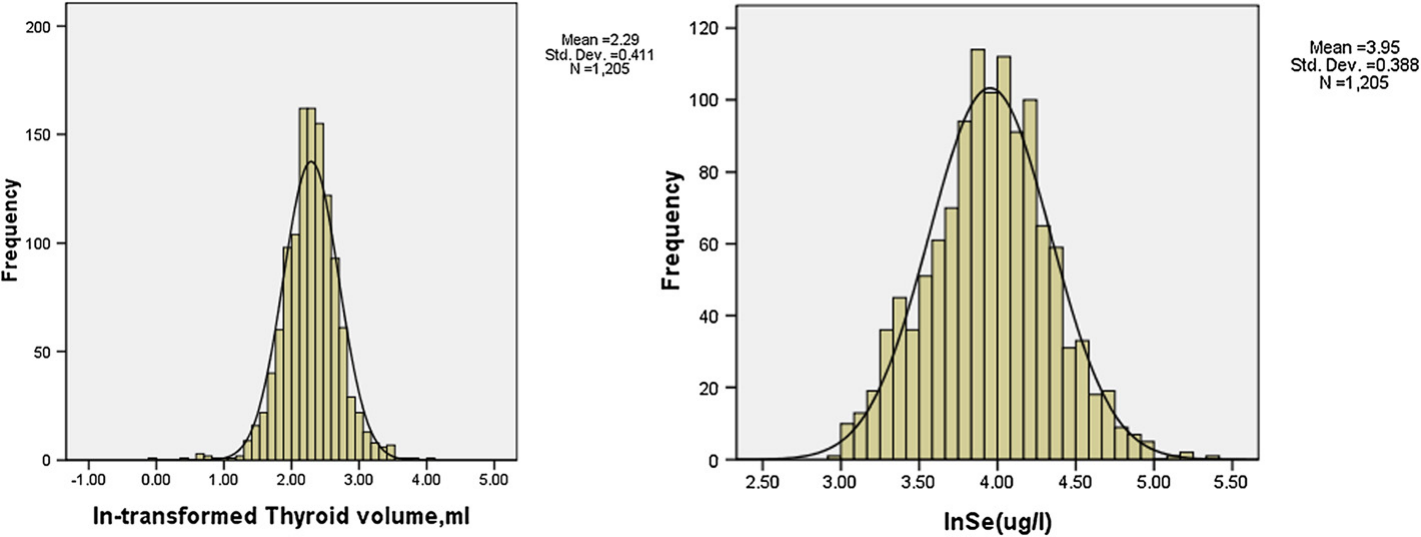
**Distribution of serum selenium (log scaled) and thyroid volumes (log scaled) for all patients.** One-Sample-Kolmogorov-Smirnov Test for ln-selenium (*P* = .638) and ln-thyroid volumes (*P* =0.046).

The serum selenium concentration and thyroid volume distribution in the 1,205 subjects were skewed, but showed a normal distribution after log transformation (Figure 1).

## Results

The median urine iodine (MUI) in the 80 school-age children was 184 μg/L. In the 1,205 subjects, the prevalences of overt hyperthyroidism, subclinical hyperthyroidism, overt hypothyroidism and subclinical hypothyroidism were 0.91%, 1.99%, 0.91% and 5.81%, respectively. Distribution of several variables among males and females are shown in Table 1. The prevalences of thyroid nodules and thyroid goiter in females were significantly higher than in males (nodules: 22.1% vs. 14.1%, *P* = .001; goiter: 11.1% vs. 5.8%, *P* = .001), as were the prevalences of positive TPOAb and TgAb (TPOAb: 15.6% vs. 5.8%, *P* = .001; TgAb: 15.7% vs. 5.3%, *P* = .001).

**Table 1:** Clinical and biological characteristics in men and women (N = 1,205)

	**Total**	**Male**	**Female**	**P**
	**n = 1205**	**n = 531 (44.07%)**	**n = 674 (55.93%)**	
Age (yr)^*^	46.31 ± 15.14	45.49 ± 15.82	46.95 ± 14.56	0.100^***^
Se (μg/L)^**^	52.63 (40.40-67.0)	51.63 (39.41-66.23)	53.14 (40.94-67.9)	0.191^***^
TV (mL)^**^	9.93 (7.71-12.57)	10.83 (8.86-13.50)	9.10 (7.26-11.57)	0.001^‡^
Nodularity N (%)	224 (18.6)	75 (14.1)	149 (22.1)	0.001^‡^
Goiter N (%)	106 (8.8)	31 (5.8)	75 (11.1)	0.001^‡^
TPOAb (+) n (%)	145 (12.0)	40 (7.5)	105 (15.6)	0.001^‡^
TgAb (+) n (%)	134 (11.1)	28 (5.3)	106 (15.7)	0.001‡
Antibody (+) n (%)	201 (16.7)	49 (9.2)	152 (22.6)	0.001‡
TSH (mU/L)	2.66 (1.81-3.66)	2.45 (1.73-3.54)	2.92 (1.9-4.13)	0.001‡‡
BMI (kg/m^2^)	22.90 ± 3.24	23.25 ± 3.03	22.62 ± 3.37	0.001^***^

Se: Selenium; TV:Thyroid volume.

Goiter: thyroid volume ≥ 14.4 mL in female, ≥18.7 L in male; TPOAb (+): TPOAb positive; TgAb (+): TgAb positive; antibody (+): TPOAb and/or TgAb positive.

^*^Mean ± standard deviation.

^**^Median (interquartile range).

^***^t test or log-transformed *t* test (selenium and thyroid volume).

^‡^Chi-square test.

^‡‡^Mann–Whitney *U* test.

### Serum selenium

In general linear univariate regression models, a positive association between serum selenium concentration and age was found (β = 0.080 ± 0.001, *P* = .006), and between the serum selenium concentration and BMI (β = 0.057 ± 0.003, *P* = .049). We found no association between serum TSH and selenium concentration (Spearman correlations, r = −0.003, *P* = .906). One hundred-six (8.8%) of the 1,205 subjects had thyroid goiters, with a median serum selenium concentration of 53.88 (IQ, 41.74-67.78) μg/L. The non-goiter group had a median serum selenium concentration of 52.54 (IQ, 40.23-66.89) μg/L. There was no significant difference in selenium levels between the two groups (log-transformed *t* test, *P* = .424). There were no differences in selenium levels between persons with nodules and without nodules (50.66 vs. 52.80, log-transformed *t*-test, *P* = .126). Similarly, there were no differences in selenium levels between positive and negative TPOAb, and positive and negative TgAb (*P* >0.05).

### Thyroid volume

Parameters estimated from the simple linear regression analysis between thyroid volume and variables that might influence thyroid volume are reported in Table 2. In the univariate analyses, there was no association between serum selenium and thyroid volume for all subjects (*P* = .977), nor in females or males (*P* > 0.05). Age, BMI, smoking, thyroid nodules, and positive TPOAb or TgAb were significantly and positively associated with thyroid volume (*P* < 0.05). TSH levels were negatively associated with thyroid volume (*P* = .001), and for every 1 mU/L TSH concentration increase, thyroid volume decreased by 0.136 mL. In the multiple linear regression model, after adjustment for age and gender, BMI, TSH, smoking, thyroid nodules, and positive thyroid autoantibodies influenced thyroid volume in the final regression model.

**Table 2:** Simple and multivariate linear regression analyses of thyroid volume* on determinants

	**β**	**95% CI**	**P**
Simple linear regression			
Age (yr)	0.065	0.000-0.003	.023
BMI (kg/m^2^)	0.149	0.012-0.026	.001
TSH (mU/L)	−0.136	−0.024-–0.010	.001
*Serum selenium (μg/L)	0.001	−0.059-0.061	.977
Smoking	0.112	0.055-0.164	.001
TPOAb positive	0.165	0.137-0.278	.001
TgAb positive	0.146	0.118-0.264	.001
Antibody positive	0.163	0.118-0.241	.001
Nodules	0.219	0.173-0.289	.001
Multivariate linear regression			
BMI (kg/m^2^)	0.126	0.009-0.023	.001
TSH (mU/L)	−0.176	−0.029-–0.015	.001
Smoking	0.120	0.064-0.169	.001
TPOAb positive	0.139	0.098-0.253	.001
TgAb positive	0.106	0.059-0.218	.001
Nodules	0.218	0.174-0.285	.001

^*^Logarithmic transformation was used to normalize the distribution.

Antibody positive:POAb and/or TgAb positive.

### Thyroid goiter

When the presence or absence of goiter was studied in our population, serum selenium was not an independent risk factor for the development of goiter (*P* = .305, OR = 1.004) (Table 3). In the univariate logistic regression model, BMI, thyroid nodules, positive TPOAb, positive TgAb, and thyroid hypoecho or uneven echo were risk factors for thyroid goiter. In the multivariate analysis, the final model retained the following variables: age, thyroid nodules, and positive thyroid autoantibodies (TPOAb and/or TgAb). There was a negative association between the presence of thyroid goiter and increasing age (β = 0.979, *P* = .006).

**Table 3:** Logistic regression analyses for risk of goiter

	**OR (95%CI)**	**P**
Univariate logistic regression		
Age (yr)	1.001 (0.998-1.014)	.871
BMI (kg/m^2^)	1.067 (1.012-1.125)	.016
TSH (mU/L)	0.997 (0.936-1.016)	.914
Serum selenium (μg/L)	1.004 (0.996-1.013)	.305
Smoking		
Heavy smoker	0.757 (0.428-1.338)	.338
Moderate smoker	0.403 (0.124-1.306)	.130
Nodules	0.230 (0.152-0.349)	.001
TPOAb positive	4.920 (3.150-7.686)	.001
TgAb positive	4.227 (2.663-6.709)	.001
Antibody positive	4.669 (3.068-7.106)	.001
Interecho		
Hypoecho	3.808 (2.281-6.356)	.001
Hyperecho	1.489 (0.187-11.992)	.703
Uneven echo	3.570 (1.648-7.731)	.001
Multivariate logistic regression		
Age (yr)	0.979 (0.964-0.994)	.006
BMI (kg/m^2^)	1.079 (1.021-1.141)	.007
Nodules	5.388 (3.359-8.641)	.001
Antibody positive	4.906 (3.148-7.647)	.001

Antibody positive: TPOAb and/or TgAb positive.

## Discussion

The median serum selenium concentration in the Chengdu community population was 52.63 (IQ, 40.40-67.00) μg/L, a relatively lower level compared with other regions. The average serum selenium was 75 μg/L in the Zhoukou dian area [13] in Beijing, 61.84 μg/L in Jinan residents [14], 132 μg/L in Canada [15], 120 μg/L in the United States [16], and 58.94 μg/L in non-goitrous areas in Turkey [17]. The differences in serum selenium concentration in different regional populations may be due to race, geographical environment, physiological status, eating habits, smoking, and the determination methodology for selenium.

Both iodine and selenium play an important role in the proper function and structure of the thyroid gland. The abnormality of iodine and selenium nutrition in populations can result in thyroid dysfunction and abnormal thyroid structure. Studies have shown that concurrent severe selenium and iodine deficiency can lead to an increase in thyroid volume and/or TSH levels [4-6], possibly because thyroid iodine deficiency stimulates the synthesis of thyroid hormones, under TSH control, leading to an increased production of hydrogen peroxide_,_ which is the electron acceptor for the thyroid peroxidase reaction in the synthesis of thyroid hormones. However, when produced excessively, hydrogen peroxide is toxic for thyroid cells [18]. As in other cells in the body, the family glutathione peroxidase, superoxide dismutase, and catalase can protect thyroid cells from hydrogen peroxide damage. On the other hand, selenium deficiency can result in lower activity of thyroid glutathione peroxidase, consequently reducing the degradation of hydrogen peroxide. Thus, a simultaneous selenium and iodine deficiency reduces the activity of thyroid glutathione peroxidase and increases hydrogen peroxide, leading to thyroid cell damage due to the highly reactive peroxides of hydrogen peroxide [19]. Selenium levels probably affect thyroid volume in areas of severe iodine deficiency, while selenium levels seem unassociated with thyroid goiter in iodine-sufficient regions. This speculation was confirmed in our study. The mean MUI levels of the school-age children in our study were 184 μg/L in our iodine-sufficient region [20]. We found that the serum selenium concentration was not associated with goiter nor did it influence thyroid volume and TSH levels. This might be because our study population lives in an area with sufficient iodine and relatively low serum selenium. The results of a Danish study [21] are consistent with our findings. Before introduction of iodine fortification, the MUI level was 57 μg/L, and the serum selenium concentration was significantly and negatively associated with a risk for an enlarged thyroid gland. However, the MUI was 100 μg/L after iodine fortification in the Danish regions, and the serum selenium concentration was no longer an independent risk factor for thyroid goiter. Therefore, to investigate the effect of serum selenium on thyroid volume and thyroid goiter, we need to determine the nutritional iodine status to eliminate the effect of different iodine levels on thyroid volume and goiter.

The status of serum selenium has diverse effects on thyroid volume and goiter in a mild iodine-deficient or (borderline) iodine-sufficient area. John et al. [12] found that when the intake of selenium was sufficient, there was no correlation between goiter and serum selenium levels even in a mild iodine-deficient area. A study in school-age children in Turkey [22] with moderate iodine deficiency, found that mean selenium was 50 μg/L, a relatively low level, and serum selenium concentration was not associated with thyroid volume, thyroid goiter, or THS levels. They considered iodine deficiency the major cause of thyroid goiter maybe because the serum selenium concentration has little influence on glutathione peroxidase and deiodinase in the thyroid gland in borderline selenium-sufficient areas. However, Keshteli et al. [23] found that selenium deficiency was one contributor of thyroid goiter in Isfahan goitrous schoolchildren in an iodine-sufficient area. Another study [24] from France, in a mild iodine-deficiency area, found a negative correlation between serum concentration and thyroid volume in 1,108 subjects. The mean serum selenium in their study was 87 μg/L, which was higher than in our population. The differences between these findings may be that the all subjects in the French cohort study were females and older (the mean age ranged from 45 to 60 years) than those in our study group. From this, it can be concluded that different serum selenium concentrations have disparate effects on thyroid volume and thyroid goiter. Serum selenium levels in the population are affected by many other factors; there is still no exact definition of selenium-deficient status. Further studies should be carried out to explore the impact of selenium concentration on thyroid volume and thyroid goiter in (borderline) iodine-sufficient areas.

The lack of an exact definition of “selenium status” may be another reason for varying results among studies in addition to variations in iodine and selenium nutritional status in different populations. In the literature, the determination of “selenium status” includes serum selenium, plasma glutathione peroxidase activity, erythrocyte glutathione peroxidase activity, urinary selenium, and hair or toenail selenium. However, the exact interrelationships among these parameters have not been determined. Some studies have shown that urinary and serum selenium concentrations are more likely to accurately reflect the presence of selenium bioavailability in the human body [25,26]. Ozata et al. [27] found that serum selenium concentration did not seem to have a role in the etiopathogenesis of goiter in Turkey in a moderate iodine-deficient area. Similarly, Brauer et al. [28] found that urinary selenium was not an independent risk factor for the development of goiter in an area with borderline iodine sufficiency and borderline selenium sufficiency. These two findings above are consistent with our results. Therefore, we conclude that selenium concentration in both serum and urine are reflective of selenium status in the body, and to some extent, indicate selenium bioavailability to the human body or thyroid gland.

## Conclusions

We can conclude that serum selenium concentrations in the Chengdu community population in China are at relatively lower levels compared with other regions. In our study population, serum selenium was neither associated with thyroid volume nor with goiter in an iodine-sufficient area. There are considerable variables that influence selenium concentrations in populations. Monitoring the selenium status of nongoitrous persons over time would help to clarify this issue.

## Competing interests

The authors declare that they have no competing interests.

## Authors’ contributions

Yang Liu collected the materials, assessed the serum selenium, extracted the data, conducted the statistical analysis and drafted the manuscript. Hui Huang conceived of the project concept, assisted with the data interpretation, and helped write and modify the manuscript. Jing Zeng helped to assay the serum selenium, and collected the data. Chengjun Sun helped to assay the serum selenium. All of the authors have read and approved the final manuscript.

## Pre-publication history

The pre-publication history for this paper can be accessed here:

http://www.biomedcentral.com/1471-2458/13/1153/prepub
